# A Novel Stopped-Flow Assay for Quantitating Carbonic-Anhydrase Activity and Assessing Red-Blood-Cell Hemolysis

**DOI:** 10.3389/fphys.2017.00169

**Published:** 2017-03-28

**Authors:** Pan Zhao, R. Ryan Geyer, Walter F. Boron

**Affiliations:** Department of Physiology and Biophysics, Case Western Reserve University School of MedicineCleveland, OH, USA

**Keywords:** pH-sensitive dyes, pyranine, phenol red, out-of-equilibrium solutions, acid-based balance, carbon dioxide, bicarbonate

## Abstract

We report a novel carbonic-anhydrase (CA) assay and its use for quantitating red-blood-cell (RBC) lysis during stopped-flow (SF) experiments. We combine two saline solutions, one containing HEPES/pH 7.03 and the other, ~1% CO_2_/44 mM ${\text{HCO}}_{3}^{-}$/pH 8.41, to generate an out-of-equilibrium CO_2_/${\text{HCO}}_{3}^{-}$ solution containing ~0.5% CO_2_/22 ${\text{HCO}}_{3}^{-}$/pH ~7.25 (10°C) in the SF reaction cell. CA catalyzes relaxation of extracellular pH to ~7.50: ${\text{HCO}}_{3}^{-}$ + H^+^ → CO_2_ + H_2_O. Proof-of-concept studies (no intact RBCs) show that the pH-relaxation rate constant (*k*_ΔpH_)—measured via pyranine fluorescence—rises linearly with increases in [bovine CAII] or [murine-RBC lysate]. The y-intercept (no CA) was *k*_ΔpH_ = 0.0183 s^−1^. Combining increasing amounts of murine-RBC lysate with ostensibly intact RBCs (pre-SF hemolysis ≅0.4%)—fixing total [hemoglobin] at 2.5 μM in the reaction cell to simulate hemolysis from ostensibly 0 to 100%—causes *k*_ΔpH_ to increase linearly. This y-intercept (0% lysate/100% ostensibly intact RBCs) was *k*_ΔpH_ = 0.0820 s^−1^, and the maximal *k*_ΔpH_ (100% lysate/0% intact RBCs) was 1.304 s^−1^. Thus, mean percent hemolysis in the reaction cell was ~4.9%. Phenol-red absorbance assays yield indistinguishable results. The increase from 0.4 to 4.9% presumably reflects mechanical RBC disruption during rapid mixing. In all fluorescence studies, the CA blocker acetazolamide reduces *k*_ΔpH_ to near-uncatalyzed values, implying that all CA activity is extracellular. Our lysis assay is simple, sensitive, and precise, and will be valuable for correcting for effects of lysis in physiological SF experiments. The underlying CA assay, applied to blood plasma, tissue-culture media, and organ perfusates could assess lysis in a variety of applications.

## Introduction

In the course of using stopped-flow (SF) absorbance spectroscopy to study the effects of inhibitors or the genetic disruption of membrane proteins on the efflux of oxygen (O_2_) from red blood cells (RBCs), we suspected that RBCs were hemolyzing in the SF reaction cell, thereby releasing hemoglobin (Hb) and leading to overestimates of the rate constants for O_2_ efflux. Moreover, different degrees of hemolysis under different experimental conditions could lead to different extents of overestimation. In our work, the computed percent hemolysis (%*H*) of RBC samples entering the SF device is far too low to influence our results appreciably, whereas the %*H* of material that eventually exits the SF device—this may occur many minutes after a single SF experiment or “shot”—is far too high to be consistent with our O_2_-efflux data. We therefore set out to develop an assay that could report %*H* within the SF reaction cell, and do so in a time domain similar to that of our O_2_-efflux experiments. Because we are concerned with Hb in the bulk extracellular fluid (BECF), we devise an assay that: [a] measures a BECF parameter; [b] uses, as far as possible, physiological reactants and products; and targets a substance that [c] is normally present exclusively in the RBC cytosol (i.e., not normally exposed to the BECF compartment) but [d] would—upon hemolysis—enter the BECF with the same time course as Hb. Two substances that fit this criterion are the high-abundance/low-activity enzyme carbonic anhydrase (CA) I and the low-abundance/high-activity CA II (Khalifah, 1971; Dodgson et al., 1988; Sly and Hu, 1995).

Since the discovery of CA by Meldrum and Roughton (1933), investigators have used several approaches for assaying the activity of this enzyme. Virtually simultaneously, Meldrum and Roughton introduced a manometric CA assay (Meldrum and Roughton, 1933), Brinkman introduced a colorimetric variant (Brinkman, 1933), and Stadie and O'Brien introduced an electrometric approach based on a platinum pH electrode (Stadie and O'Brien, 1933). In 1948, Wilbur and Anderson replaced platinum with a glass pH electrode (Wilbur and Anderson, 1948). All of these approaches are intrinsically slow and thus require that the preparation be a low temperature (0°C). After the introduction of stopped-flow devices by Chance in 1950 (Chance, 1950), several investigators used stopped-flow absorbance or fluorescence spectroscopy to study the kinetics of CA (Gibbons and Edsall, 1963, 1964; Ho and Sturtevant, 1963; Kernohan, 1964, 1965; Khalifah, 1971; Wistrand et al., 1975; Pocker and Bjorkquist, 1977; Crandall and O'Brasky, 1978; DeGrado et al., 1982; Sanyal et al., 1982; Baird et al., 1997; Shingles and Moroney, 1997; Wang et al., 2010). These early reports had in common the mixing of two solutions having dissimilar CO_2_/${\text{HCO}}_{3}^{-}$/pH status, and the monitoring of the subsequent time course of at least one parameter from a pH-sensitive dye. Except for one case (Shingles and Moroney, 1997), these earlier papers provided only rudimentary experimental detail, raising the concern that at least one of the two initial solutions had a pH sufficiently extreme as to be incompatible with living cells. Later investigators introduced CA assays based on continuous-flow calorimetry (Kernohan and Roughton, 1968), the release of ^14^CO_2_ (Stemler, 1993), and the exchange of ^18^O-labeled CO_2_/${\text{HCO}}_{3}^{-}$ as measured by mass spectrometry (Itada and Forster, 1977). Although the authors' main objective in the ^18^O-exchange study was to measure the CA activity of intact RBCs, they recognized that their assay is very sensitive to small degrees of CA released by hemolysis. Nevertheless, none of these approaches is easily translated to a SF platform as a method for assessing hemolysis in real time.

Here, we describe novel methods for assaying CA activity in a SF device, and then extending these assays to assess RBC hemolysis. Our CA assay exploits earlier work from our laboratory to generate out-of-equilibrium (OOE) CO_2_/${\text{HCO}}_{3}^{-}$ solutions (Zhao et al., 1995) with virtually any combination of [CO_2_], [${\text{HCO}}_{3}^{-}$], and pH, even somewhat beyond the pathophysiological range of values. In the present study, we combine two dissimilar CO_2_/${\text{HCO}}_{3}^{-}$ solutions—solution (A) 0% CO_2_/0 ${\text{HCO}}_{3}^{-}$/pH 7.03 and solution (B) 1% CO_2_/44 mM ${\text{HCO}}_{3}^{-}$/pH 8.41—to create in the reaction cell of an SF device a predetermined initial OOE state—0.5% CO_2_/22 mM ${\text{HCO}}_{3}^{-}$/pH ~7.25—in which the pH is far too low for the predetermined [CO_2_]/[${\text{HCO}}_{3}^{-}$] ratio. Thus, the system spontaneously undergoes the reactions ${\text{HCO}}_{3}^{-}+{\text{H}}^{+}\overset{\text{rapid}}{\to }{\text{H}}_{2}{\text{CO}}_{3}\overset{\text{slow}}{\to }{\text{CO}}_{2}+{\text{H}}_{2}\text{O},$ causing pH in the SF reaction cell to rise to ~7.50. Because CA catalyzes the reaction ${\text{HCO}}_{3}^{-}+{\text{H}}^{+}\overset{\text{CA}}{\to }{\text{CO}}_{2}+{\text{H}}_{2}\text{O},$ (effectively bypassing the slow H_2_CO_3_ dehydration), we can use the time course of pH, reported by the fluorescent dye pyranine (also known as HPTS for 8-hydroxypyrene-1,3,6-trisulfonic acid, trisodium salt) or the non-fluorescent dye phenol red, to compute CA activity. We validate this technique on purified bovine CA II (bCAII) and hemolysates of mouse blood, and then apply it to ostensibly intact murine RBCs, where we find that the actual hemolysis is ~4.9%. Given the sensitivity, precision, and ease of our methodology, this approach could be valuable for assessing CA release (i.e., lysis) from virtually any membrane-bound structure during SF experiments, or for detecting CA release from fluid that was previously in contact with living cells.

## Materials and methods

### Ethical approval

Protocols for housing and handling of mice were approved by the Institutional Animal Care and Use Committee at Case Western Reserve University.

### Solutions

The compositions of solutions are shown in Table 1. For the CA assay, we combined solution A with solution B in the SF device to obtain the initial OOE solution (“Mix”) in the reaction cell. To achieve the desired pH, we titrated solutions with NaOH or HCl either at room temperature (RT, for RBC washing solution) or at 10°C (for pH calibration solution and OOE A and B solutions). pH measurements were recorded on a portable pH meter (model A121 Orion Star, Thermo Scientific, Beverly, MA) fitted with a pH electrode (Ross Sure-Flow combination pH Electrode, Thermo Scientific) at RT or at 10°C. For all work at 10°C—both pH titration of solutions and the actual experiments—we used a refrigerated, constant-temperature, shaker water bath (model RWB 3220, Thermo Fisher Scientific Inc., Asheville, NC) and several Telesystem magnetic stirrers (Thermo Fisher Scientific). Beakers containing the pH calibration buffers (pH at 6, 8, and 10, Fisher Scientific, Fair Lawn, NJ), the solutions to be titrated, and the pH electrode in its storage solution (Beckman Coulter, Inc. Brea, CA) were equilibrated, as appropriate, at either RT or 10°C. Osmolality was measured using a vapor pressure osmometer (Vapro 5520; Wescor, Inc., Logan, UT), and adjusted upward if necessary by the addition of NaCl.

**Table 1:** **Physiological solutions**.

**Component or parameter**	**RBC washing solution**	**pH calibration solution for pyranine**	**OOE solution for CA assay with pyranine**			**pH calibration solution for phenol red**	**OOE solution for CA assay with Phenol Red**		
			**A**	**B**	**Mix^¶^**		**A**	**B**	**Mix^¶^**
NaCl (mM)	92.5	150	140	116	128	151	142	116	128
KCl (mM)	0	3	3	3	3	3	3	3	3
CaCl_2_ (mM)	0.01	1	2	0	1	1	2	0	1
Na_2_HPO_4_ (mM)^*^	46.98	0	0	0	0	0	0	0	0
NaH_2_PO_4_ (mM)^*^	11.02	0	0	0	0	0	0	0	0
HEPES (mM)^†^	0	8	16	0	8	7	14	0	7
NaHCO_3_ (mM)^‡^	0	0	~0	44	22	0	~0	44	22
CO_2_ (%)	0	0	~0	~1	0.5	0	~0	~1	0.5
pH	~7.40^*^	7.50^†^	7.03^†^	8.41^‡^	~7.25	7.50^†^	7.03^†^	8.41^‡^	~7.30
Pyranine (μM)^§^	0	1 or 0	0	2 or 0	1 or 0	0	0	0	0
Phenol red (mM)^§^	0	0	0	0	0	1 or 0	0	2 or 0	1or 0
bCAII, lysate, RBCs	0	0	++ or 0	0	+ or 0	0	++ or 0	0	+ or 0
ACZ	0	0	0	++ or 0	+ or 0	0	0	0	0
Temperature (°C)	RT	10	10	10	10	10	10	10	10
Osmolality (mOsm)	~300	~295	~295	~300	~298	~295	~295	~300	~298

* *The ratio [${\text{HPO}}_{\text{4}}^{=}$]/[H_2_${\text{PO}}_{4}^{-}$] determines the pH at RT (~22°C)*.

† *For the pH calibration solution, we titrated HEPES free acid (pK ~7.5) to pH 7.50 with NaOH, and then in some aliquots added either HCl or more NaOH to achieve pH-values from 5.50 to 8.50 at 10°C. After the titration, we added pyranine or phenol red to equal concentrations in each solution. For OOE solution A, we titrated HEPES free acid to pH 7. 03 with NaOH*.

‡ *The addition of ${\text{HCO}}_{3}^{-}$ generates some CO_2_ and ${\text{CO}}_{3}^{=}$; this mixture determined the final pH at 10°C*.

§ *[Pyranine] was present at a concentration of 1 μM in the reaction cell (to obtain pH data) or 0 μM (to obtain background data)*.

§ *[Phenol Red] was present at a concentration of 1 mM in the reaction cell (to obtain pH data) or 0 mM (to obtain background data)*.

¶ *The values in this pyranine column are those at the instant of mixing solutions A and B. The solution is out of equilibrium because the pH of 7.25 is far too low, given [${\text{HCO}}_{3}^{-}$] = 22 mM and CO_2_ = 0.5%*.

¶ *The values in this Phenol Red column are those at the instant of mixing solutions A and B*.

### Stopped-flow fluorescence spectroscopy

#### Data acquisition and analysis

We rapidly combined solutions A and B using the SX-20 stopped-flow apparatus (Applied Photophysics, Leatherhead, UK). We excited the pH-sensitive fluorescent dye pyranine (H348, Invitrogen, Eugene, OR, USA; see Avnir and Barenholz (2005) using an excitation wavelength of 460 nm (pH-sensitive wavelength), or of 415 nm (pH-independent isosbestic point), while monitoring total fluorescence emission using a 488 nm long-pass filter. The sampling period of the SF device was 12.5 μs. Because the output of the device was 1 data point every 0.1 s, each data point represents (10^−1^ s)/(12.5 × 10^−6^ s) = 8,000 samples. Our duration of data collection ranged from 20 s (i.e., 200 data points) for rapid reactions (i.e., high CA activity, A) to 200 s (i.e., 2000 data points) for slow reactions (e.g., the uncatalyzed reaction, where A = 1, the minimum value).

For each new experimental sample of solutions A and B (see Table 1), we began by performing six SF shots to ensure that the new solutions were loaded into the reaction cell, and then sequentially acquired four time courses. First, we acquired two time courses in which solution B contained no dye (see Table 1), one a time course of *I*_Background, 460_ during one stopped-flow shot while exciting at 460 nm, and then a time course of *I*_Background, 415_ during a second shot while exciting at 415 nm. Then we replaced solution B with one that had an identical composition except for the inclusion of dye (see Table 1), performed six additional SF shots, and then acquired two time additional courses, one while exciting at 460 nm to yield *I*_Total, 460_, and another at 415 nm to yield *I*_Total, 415_. After correcting for background, we obtained for each experimental sample the time course of the ratio (*I*_460_/*I*_415_) = [(*I*_Total, 460_ − *I*_Background, 460_)/(*I*_Total, 415_ − *I*_Background, 415_)], which we converted to the time course of pH as described below. For some samples, solution B contained acetazolamide (see Table 1). After each experimental sample—for which we acquired four sequential time courses as outlined above—we extensively flushed (2 × 2.5 ml in each syringe) both the “A” and “B” lines of the SF device with OOE “A” buffer (for the “A” line) or “B” buffer (for the “B” line). The exception was that we did not flush line “A” between samples in a sequence of ascending [bCAII] in **Figure 2**, or ascending RBC lysate in **Figure 3**.

#### Calibration of dye

We chose pyranine (HPTS) as our fluorescent pH indicator because others have found it suitable for monitoring extracellular pH; its favorable properties include a low rate of permeation across cell membranes (Shingles and Moroney, 1997; Avnir and Barenholz, 2005; Han and Burgess, 2010). We calibrated the pH indicator dye pyranine at 10°C by mixing, in the SF device, two identical solutions that were either the pH-7.50 “pH calibration solution for pyranine” listed in Table 1, or variants thereof obtained by titrating the pH as outlined in a footnote to Table 1. For each pH-value X, we computed (*I*_460_/*I*_415_)_pHx_ as described in the previous section. Using an approach described previously for another pH-sensitive fluorophore (Boyarsky et al., 1988), we fitted the following theoretical titration curve to our experimental data:

$$\frac{{\left(I_{460}/I_{415}\right)}_{\text{pHx}}}{{\left(I_{460}/I_{415}\right)}_{\text{pH}7.5}}=1+b\left[\frac{10^{\left(\text{pHx}-\text{pK}\right)}}{1+10^{\left(\text{pHx}-\text{pK}\right)}}-\frac{10^{\left(7.5-\text{pK}\right)}}{1+10^{\left(7.5-\text{pK}\right)}}\right],$$

which normalizes the data to the value observed at pH 7.5, and forces the function to have a value of unity at this pH. Here, *b* is the difference between the maximal and minimal asymptotic values of (*I*_460_/*I*_415_)_pHx_/(*I*_460_/*I*_415_)_7.5_.

**Figure 1:**
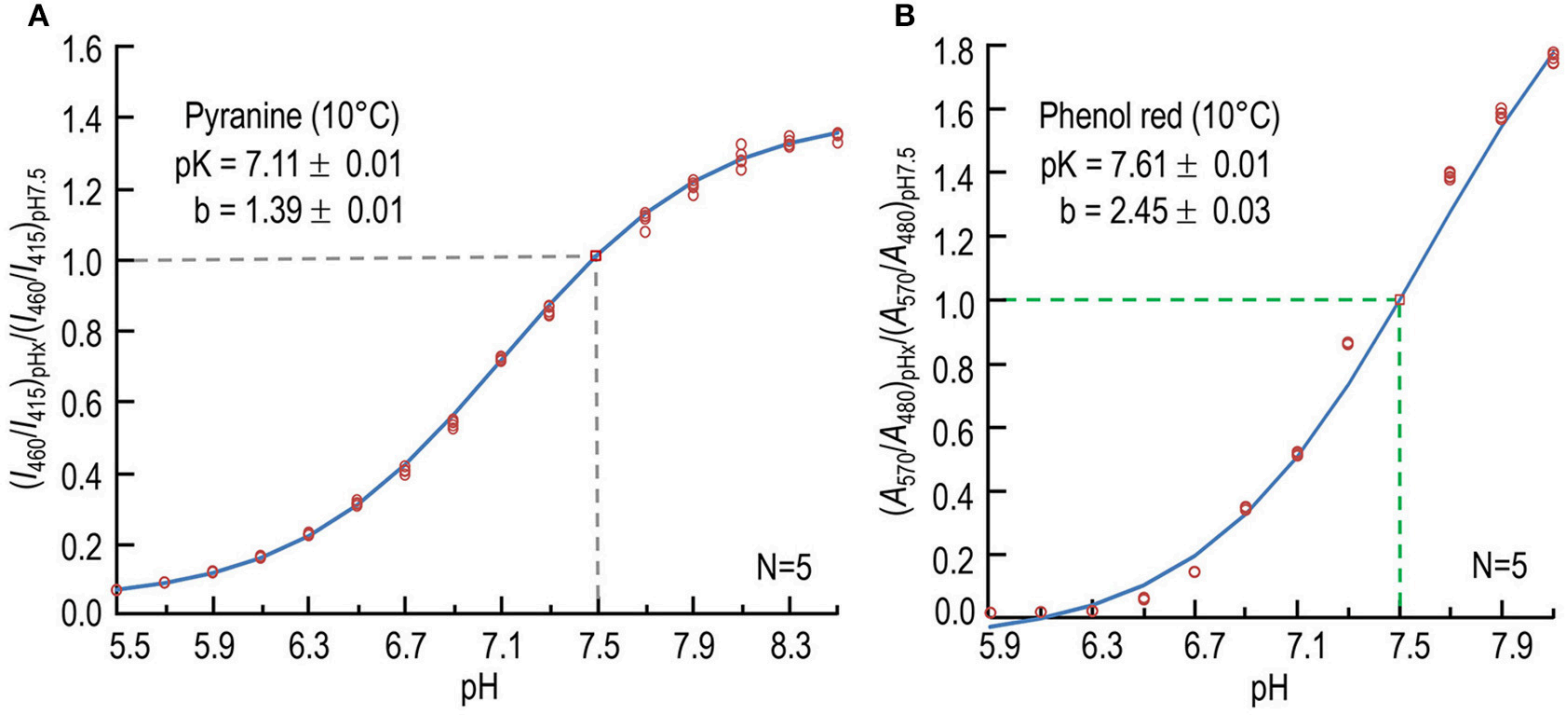
**Calibration of pH-sensitive dyes at 10°C. (A)** Dependence of normalized fluorescence-excitation ratio of pyranine on pH. For each data point at one of 16 pH-values (pH_x_), we record the time course of fluorescence emission (≥488 nm) in the stopped-flow device separately at excitation wavelengths of 460 and 415 nm, first in the absence and then in the presence of dye, and obtain the background-subtracted ratio (I_460_/I_415_)_pHx_, as described in Section Materials and Methods. We then divide each (I_460_/I_415_)_pHx_ by the value observed at pH 7.50 in that experiment to obtain the normalized fluorescence-excitation ratio (I_460_/I_415_)_pHx_/(I_460_/I_415_)_pH7.5_. The value 7.11 is the best-fit value for pK, and 1.39 is the best-fit value for the difference between the maximal and minimal asymptotic values of (*I*_460_/*I*_415_)_pH_x__/(*I*_460_/*I*_415_)_7.5_. The data are from five sets of experiments. **(B)** Dependence of normalized absorbance ratio of phenol red on pH. For each data point at one of 12 pH-values (pH_x_), we record absorbance separately at the pH-sensitive wavelength of 570 nm and at the isosbestic wavelength of 480 nm. After subtracting the background at each incident wavelength, we obtain the ratio (A_570_/A_480_)_pHx_. We then normalize each (A_570_/A_480_)_pHx_ to the value observed at pH 7.50 in that experiment, as described in Section Materials and Methods. The data are from five sets of experiments. In both **(A,B)**, we show each individual point, some of which overlie other. *N* is the number of experiments in each experiment, we obtained data for each pH. The curves through the points—and the pK and *b*-values (±*SD*)—are the result of non-linear least-squares calculations.

We used an iterative, non-linear least-squares method (Boyarsky et al., 1988) to determine *b* and pK. Figure 1A shows a plot of the data from five sets of experiments, as well as the best-fit curve. The best-fit values were 7.11 ± 0.01 (*SD*) for the pK, and 1.39 ± 0.01 (*SD*) for *b*. We used these values of pK and *b*—obtained at 10°C—and the values for ratio of (*I*_460_/*I*_415_) in each experiment to calculate pH. Others have reported pK-values for pyranine of 7.24 at RT (Avnir and Barenholz, 2005) and ~7.3 at 25°C (Han and Burgess, 2010).

### Stopped-flow absorbance spectroscopy

#### Data acquisition and analysis

Using an approach similar to that outlined above for stopped-flow fluorescence spectroscopy, we used stopped-flow absorbance spectroscopy with the non-fluorescent pH-sensitive dye phenol red (P4633, Sigma-Aldrich, St. Louis, MO) to record absorbance at wavelengths of 570 nm (pH-sensitive wavelength) and 480 nm (pH isosbestic wavelength; Hollingworth and Baylor, 1990). The sampling period was 12.5 μs, and the duration of data collection ranged from 20 to 200 s.

For each experimental sample, we acquired two time courses in the absence of dye (Table 1), *A*_Background, 570_ and *A*_Background, 480_, and also two time courses in the presence of dye (Table 1), *A*_Total, 570_ and *A*_Total, 480_. For each experimental sample, we obtained the time course of the ratio (*A*_570_/*A*_480_) = [(*A*_Total, 570_ − *A*_Background, 570_)/(*A*_Total, 480_ − *A*_Background, 480_)], which we converted to the time course of pH as described below.

#### Calibration of dye

We chose phenol red as our non-fluorescent pH indicator because others have found it suitable for long-term monitoring of pH in cell- or tissue-culture media. We calibrated the pH indicator phenol red at 10°C by mixing, in the SF device, two identical solutions that were either the pH-7.50 “pH calibration solution for Phenol red” listed in Table 1, or variants thereof at different pH-values (see footnote to Table 1). For each pH-value X, we computed (*A*_570_/*A*_480_)_pHx_, using an equation similar to that described above for pyranine:

$$\frac{{\left(A_{570}/A_{480}\right)}_{\text{pHx}}}{{\left(A_{570}/A_{480}\right)}_{\text{pH}7.5}}=1+b\left[\frac{10^{\left(\text{pHx}-\text{pK}\right)}}{1+10^{\left(\text{pHx}-\text{pK}\right)}}-\frac{10^{\left(7.5-\text{pK}\right)}}{1+10^{\left(7.5-\text{pK}\right)}}\right].$$

Figure 1B shows a plot of the data from five sets of experiments, as well as the best-fit curve. The best-fit values, obtained using an iterative, non-linear least-squares method (Boyarsky et al., 1988), were 7.61 ± 0.01 (*SD*) for the pK, and 2.45 ± 0.03 (*SD*) for *b*. We used these values of pK and *b*—obtained at 10°C—and the values for ratio of (*A*_570_/*A*_480_) in each experiment to calculate pH. Others have reported the pK of phenol red to be 7.67 at 25°C (Sendroy and Rodkey, 1961), and 7.73 at 16–17°C (Hollingworth and Baylor, 1990).

### Carbonic anhydrase assay

For some experiments, we obtained purified bCAII, isolated from erythrocytes (C2522, Sigma-Aldrich, St. Louis, MO), and resuspended it in 0.2% bovine serum albumin at a concentration of 1 mg/mL. We added varying amounts of bCAII to establish concentrations from 0.5 to 8 μg/mL in solution A (Table 1). Rapid mixing with solution B (containing 2 μM pyranine) at 10°C in the SF reaction cell initiates the reactions $\text{HC}{\text{O}}_{3}^{-}+{\text{H}}^{+}\to {\text{H}}_{2}\text{C}{\text{O}}_{3}\to \text{C}{\text{O}}_{2}+{\text{H}}_{2}\text{O and HC}{\text{O}}_{3}^{-}+{\text{H}}^{+}\overset{\text{CA}}{\to }\text{C}{\text{O}}_{2}+{\text{H}}_{2}\text{O},$ causing pH to rise exponentially. Under stopped-flow conditions, we exploited the fluorescence of pyranine to monitor this pH trajectory as described above. In other experiments, instead of adding bCAII to solution A, we added murine RBC lysate (described below), murine RBCs (described below), or mixtures of the two. In some of the lysate and RBC experiments, we instead exploited the absorbance of phenol red.

Regardless of the dye used, we fitted the pH time course with the equation

$$\begin{array}{l} \text{pH}(t)=A-Be^{-(k_{\Delta pH})t}, \end{array}$$

where *t* is time, *A* is the final (equilibrated) value of pH, *B* is the pH range, and *k*_ΔpH_ is the rate constant of the pH relaxation. We obtained *A, B*, and *k*_ΔpH_ using a non-linear least-squares method.

### Blood collection

Prior to blood collection, a 1-mL syringe and attached 23-gauge needle were rinsed with 0.1% sodium heparin (H4784, Sigma-Aldrich). Adult C57/BL6 wild type (WT) mice (8–16 weeks old) were sacrificed by cervical dislocation and blood was immediately collected by the cardiac-puncture method (Parasuraman et al., 2010) using the aforementioned syringe and needle. The blood was transferred to a 1.5-mL tube, centrifuged in a Beckman Microfuge 16 Microcentrifuge (Beckman, Brea, CA) at 600 × g for 10 min and the resulting supernatant #0 and buffy coat were removed. To remove residual extracellular Hb, the pelleted RBCs were resuspended (“resuspended RBCs”) in RBC washing solution (Table 1) to a ~5–10% hematocrit (Hct), and centrifuged at 600 × g for 5 min. The supernatant from this centrifugation is supernatant #1. This process was repeated 3×, with an estimation of percent hemolysis (see below) performed at each step. After these four washes, RBCs were resuspended in RBC washing solution to a final Hct of 25–30%, and maintained for up to ~5 h on ice for experiments performed that day.

We computed the Hb concentration, [Hb], using a novel least-square's approach, based on Beer's law. It is well-known that pure Hb has an absorbance at 650 nm (*A*_650_) of ~0 (Philo et al., 1981; Barlow et al., 1992; Hernández et al., 2009), and we found that *A*_650_ is likewise ~0 in RBC hemolysates (data not shown; preparation described in next section). When we compared absorbance spectra of ostensibly intact RBCs with those of RBC lysates of equal [Hb] value, we found that they were virtually identical except that the spectra for ostensibly intact RBCs were displaced upward by almost exactly *A*_650_ (not shown). Therefore, to compute [Hb]—regardless of whether we were dealing with samples of ostensibly intact RBCs or RBC lysate—we determined *A*_560_, *A*_576_, and *A*_650_ on a Beckman Coulter 730 Life Science UV/Vis Spectrophotometer (Beckman, Brea, CA), using the following equation (see following section for derivation):

$$[\text{Hb}](\text{M})=\frac{{\left(A_{560}-A_{650}\right)}^{2}+{\left(A_{576}-A_{650}\right)}^{2}}{\ell \varepsilon_{560}\left(A_{560}-A_{650}\right)+\ell \varepsilon_{576}\left(A_{576}-A_{650}\right)},$$

where *l* is the pathlength of 1 cm, the molar extinction coefficient for oxy-hemoglobin at 560 nm (ε_560_) is 32,613.2 cm^−1^ M^−1^, and ε_576_ is 55,540 cm^−1^ M^−1^ (Prahl, 1998).

After the initial centrifugation of whole blood, we estimated percent hemolysis after determining [Hb] in (a) the blood plasma (i.e., supernatant #0; see definition, above), and (b) in the pelleted RBCs after their resuspension in RBC washing solution, using the equation:

$$\begin{array}{l} \%H\text{ before first wash}\\ \;=\frac{{\left[\text{Hb}\right]}_{\text{Supernatant}\#0}\times V_{\text{Supernatant}\#0}}{\begin{array}{c} \left({\left[\text{Hb}\right]}_{\text{Supernatant}\#0}\times V_{\text{Supernatant }\#0}\right)+\\ \left({\left[\text{Hb}\right]}_{\text{Resuspended RBCs}}\times V_{\text{Resuspended RBCs}}\right) \end{array}}, \end{array}$$

where *V* is volume. In **Figure 5A**, the resulting value is plotted as “Number of washes of RBCs” = 0.

After each step *i* of RBC washing, we estimated %*H* hemolysis by determining [Hb] in the supernatant #*i* and the resuspended RBCs:

$$\begin{array}{l} \%H\text{ after wash }\#i\\ \;=\frac{{\left[\text{Hb}\right]}_{\text{Supernatant}\#i}\times V_{\text{Supernatant }\#i}}{\begin{array}{c} \left({\left[\text{Hb}\right]}_{\text{Supernatant}\#0}\times V_{\text{Supernatant}\#0}\right)+\\ \left({\left[\text{Hb}\right]}_{\text{Resuspended RBCs}}\times V_{\text{Resuspended RBCs}}\right) \end{array}}, \end{array}$$

where *V* is volume. In **Figure 5A**, the resulting values are plotted as “Number of washes of RBCs” = 1, 2, 3, and 4. Because a tiny fraction of RBCs may hemolyzed with each wash, the preceding equation may slightly underestimate the true %*H*.

### Derivation of equation for hemoglobin concentration

According to the Beer-Lambert law, the hemoglobin concentration is [Hb] = *A*_λ_/ℓε_λ_, where *A*_λ_ is absorbance at wavelength λ, ℓ is pathlength (cm), and ε_λ_ is the molar extinction coefficient (cm^−1^ M^−1^).

To determine the concentration (*C*) from the following equation:

$$\begin{array}{l} {}[\text{Hb}](\text{M})=C=\frac{A}{\ell \varepsilon }, \end{array}$$

we used a least-squares method. To avoid inaccuracies caused by dividing by ε, which varies substantially, consider

$$\begin{array}{l} \frac{A}{C}-\ell \varepsilon =0. \end{array}$$

If we denote

$$\begin{array}{l} \bar{C}\;=\;\frac{1}{C} \end{array}$$

then

$$\begin{array}{l} A\bar{C}-\ell \varepsilon =0. \end{array}$$

In the present study, we obtain three absorbance values—*A*_560_ (560 nm is a local valley for oxygenated Hb), *A*_576_ (576 nm is a local peak), and *A*_650_ (650 nm is O_2_ independent)—from which we derive *A*_1_ = *A*_560_ − *A*_650_ and *A*_2_ = *A*_576_ − *A*_650_. Because each derived *A*-value has a corresponding ε-value, we have two data points (*A*_1_, ε_1_) and (*A*_2_, ε_2_). The objective of the least-squares method is to find the best-fit value of *C* that minimizes the value of the following function:

$$F={\left(A_{1}\bar{C}-\ell \varepsilon_{1}\right)}^{2}+{\left(A_{2}\bar{C}-\ell \varepsilon_{2}\right)}^{2}.$$

This minimum occurs when

$$\frac{dF}{d\bar{C}}=0,$$

which occurs when

$$\begin{array}{l} 2A_{1}(A_{1}\bar{C}-\ell \varepsilon_{1})+2A_{2}(A_{2}\bar{C}-\ell \varepsilon_{2})=0,\\ \;\bar{C}(A_{1}^{2}+A_{2}^{2})-\ell (A_{1}\varepsilon_{1}+A_{2}\varepsilon_{2})=0,\\ \;\bar{C}=\frac{\ell (A_{1}\varepsilon_{1}+A_{2}\varepsilon_{2})}{A_{1}^{2}+A_{2}^{2}}. \end{array}$$

Thus, the concentration (*C*) is:

$$C=\frac{A_{1}^{2}+A_{2}^{2}}{\ell (A_{1}\varepsilon_{1}+A_{2}\varepsilon_{2})}.$$

### Simulated hemolysis

An RBC lysate was produced by osmotic lysis of 20 μL of freshly prepared, packed mouse RBCs (see above) in Milli-Q H_2_O (Milli-Q® Integral Water Purification System, EMD Millipore Corporation, Billerica, MA) of ~1:8 dilution, followed by centrifugation at 15000 × g for 5 min in a Beckman Microfuge 16 at RT. The supernatant (cleared of cellular debris) then was removed and the hemolysate was transferred to a clean 1.5 mL tube for a spectroscopic determination of [Hb] as described above.

We achieved simulated degrees of hemolysis (Table 2, column 1), ranging from 0% (an apparent value) to 100% (an actual value) by combining different proportions of (a) freshly prepared, ostensibly intact RBCs (Table 2, column 2) and (b) a lysate (representing 100% lysis; Table 2, column 3), while maintaining the total [Hb] at 2.5 μM in the SF reaction cell.

**Table 2:** **Establishing the fraction of apparent hemolysis**.

**% Apparent hemolysis (%)**	Intact RBCs^*^(%)	RBC hemolysate^*^ (%)
0	100	0
5	95	5
10	90	10
25	75	25
50	50	50
100	0	100

* *100% refers to 2.5 μM hemoglobin in the reaction cell*.

### Analysis of data

We report results as mean ± *SD*. We analyze data using two-tailed unpaired student's *t*-test or two-tailed paired student's *t*-test, considering *P* < 0.05 as significant. In **Figure 5A**, we also applied the Holm-Bonferroni correction for the 10 comparisons and using α = 0.05 (Holm, 1979).

## Results

We developed a novel CA assay, based on the first use of out-of-equilibrium CO_2_/${\text{HCO}}_{3}^{-}$ solutions in a stopped-flow device. OOE technology makes it possible to generate, for a brief period of time, solutions in which CO_2_, ${\text{HCO}}_{3}^{-}$ and H^+^ are predictably out of equilibrium, even though each of their concentrations may have near-physiological values (Zhao et al., 1995). We use the fluorescent dye pyranine and SF fluorescence spectroscopy for most of our analyses. However, in some experiments, we use the dye phenol red and SF absorbance spectroscopy. We choose an SF temperature of 10°C to match the condition of our parallel O_2_ study, and at the same time maintain a suitable CA catalytic rate. We choose the pH to be ~7.25 at the instant of mixing in the SF reaction cell. Over the ensuing seconds, the net reaction ${\text{HCO}}_{3}^{-}$ + H^+^ → CO_2_ + H_2_O causes pH to rise exponentially to ~7.50. We estimate^1^ that, during this time, [${\text{HCO}}_{3}^{-}$] falls from 22.0 to 21.16 mM, and that [CO_2_] rises by the same amount, from 0.23 mM (i.e., 0.5% CO_2_) to 1.06 mM. Because the Hct in the SF reaction cell is ~0.15% (see below), any traffic of CO_2_ or ${\text{HCO}}_{3}^{-}$ across the RBC membrane would have only trivial effects on extracellular composition. We assume that changes in intracellular composition would not affect the degree of hemolysis.

### CA assay on purified bovine CA II

**Figure 2:**
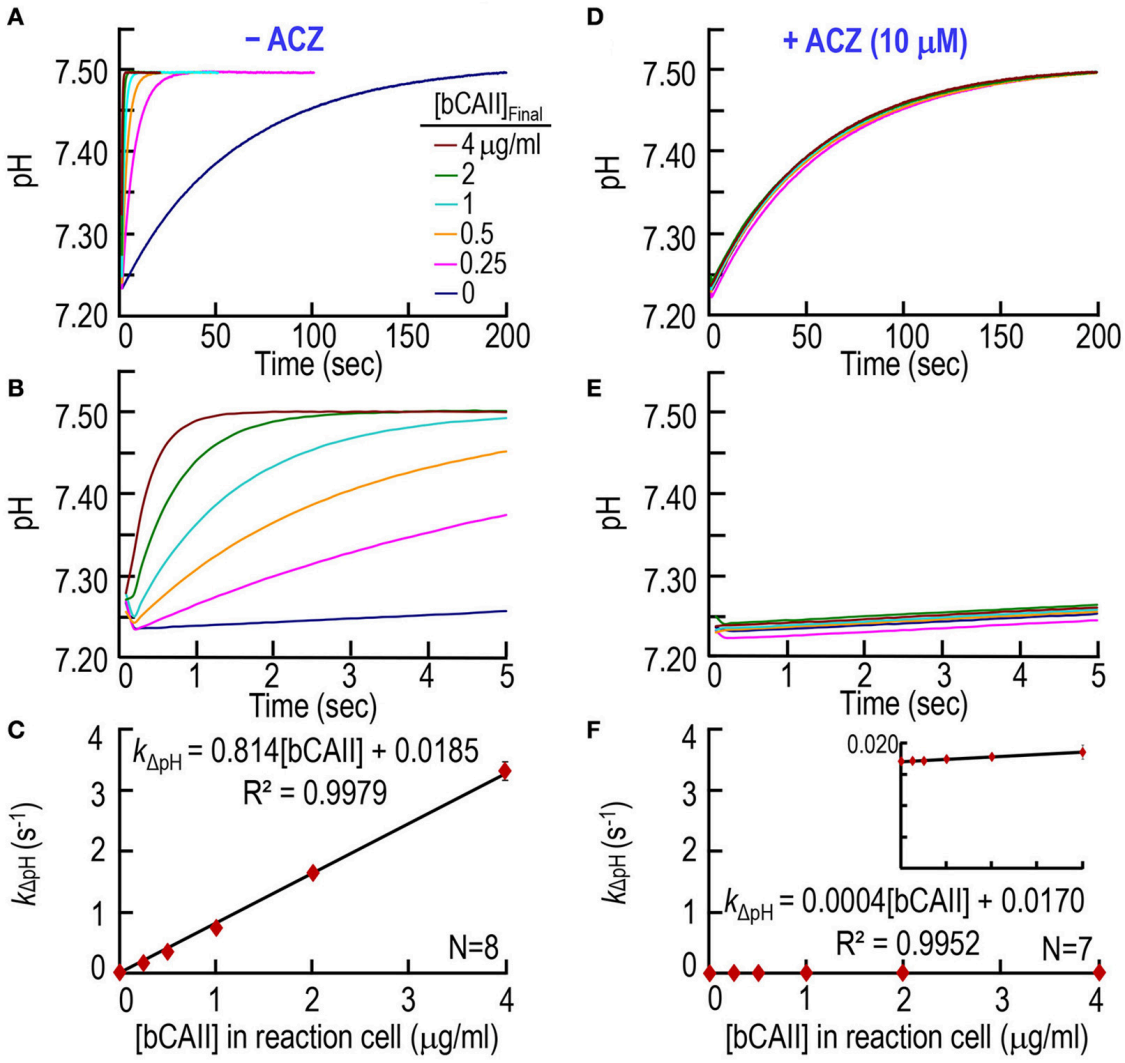
**CA assays on purified bovine CA II. (A)** Time courses of pH from 0 to 200 s, for concentrations of bCAII ranging from 0 to 4 μg/ml in the reaction cell. We added bCAII to solution A (Table 1) and derived pH-values from the fluorescence of pyranine, added to solution B. **(B)** Time courses from 0 to 5 s, representing the same records as in **(A)**. **(C)** Dependence of the rate constant of the pH change on [bCAII]_RxCell_. We obtained *k*_ΔpH_-values from non-linear least-squares curve fits of data like those in **(A,B)**. **(D)** Effect of acetazolamide on time courses of pH from 0 to 200 s for [bCAII]_RxCell_ ranging from 0 to 4 μg/ml. These experiments are like those in **(A)**, except that we added 20 μM ACZ to solution B (Table 1). **(E)** Time courses from 0 to 5 s, representing the same records as in **(D)**. **(F)** Dependence of *k*_ΔpH_ on [bCAII]_RxCell_ in the presence of ACZ. Each data point represents mean ± *SD* (error bar not shown if smaller than the symbol). *N* represents the number of independent experiments, each covering six concentrations (0–4 μg/ml).

First, we test our CA assay on purified bCAII, over a range of [bCAII] values. The pyranine dye is in the syringe opposite to the one containing bCAII (Table 1). With no added CA (0 μg/ml), pH rises very slowly, reaching ~7.50 in ~200 s (Figure 2A, lowest curve). As we increase the bCAII concentration in the SF reaction cell (i.e., [bCAII]_RxCell_ = 0.25, 0.5, 1, 2, and 4 μg/ml), the equilibration speeds greatly. Figure 2B shows the same data as in Figure 2A, but over the first 5 s. For each [bCAII] in Figures 2A,B —and many other similar experiments—we calculate the rate constant of the pH relaxation, using a least-squares approach to fit an exponential curve to each set of (pH, *t*) coordinates that describes the exponential increase in pH over time. Figure 2C summarizes the dependence of the rate constant *k*_ΔpH_ on [bCAII] at 10°C, and shows that graded increases in [bCAII] cause *k*_ΔpH_ to rise linearly. The y-intercept of the line of best fit, 0.0185 s^−1^, is the rate constant of the uncatalyzed chemical reactions that occur during the pH relaxation. The slope of the line, 0.8143 (s^−1^)/(μg/ml), is a measure of the specific activity of the bCAII.

Next, we studied the effect of CA blocker acetazolamide (ACZ) on bCAII enzymatic activity, adding ACZ to the syringe opposite to the one containing bCAII (Table 1) to achieve a [ACZ]_RxCell_ in the reaction cell of 10 μM. Figures 2D,E, as expected, show that ACZ virtually collapses all the pH relaxations for the bCAII experiments (0.25–4 μg/ml) onto the curve describing no added bCAII. The summary in Figure 2F shows that, over the entire range of [bCAII] values, ACZ reduces *k*_ΔpH_ to nearly the uncatalyzed value.

### CA assay on mouse RBC hemolysate

**Figure 3:**
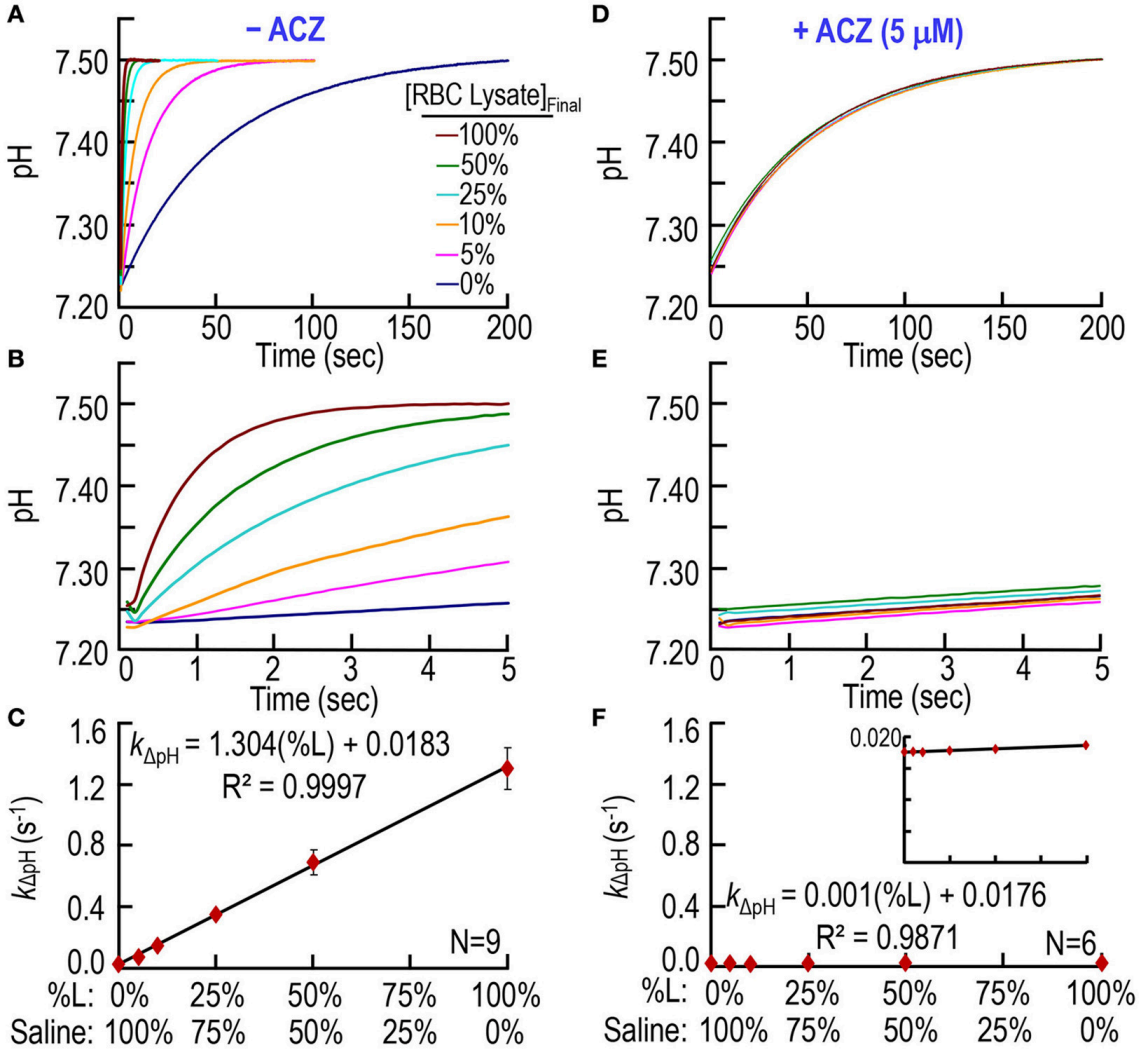
**CA assays on lysate from mouse RBCs. (A)** Time courses of pH from 0 to 200 s, for different relative amounts of hemolysate. We added RBC lysate to solution A (Table 1) and derived pH-values from the fluorescence of pyranine, added to solution B. We created all solutions by mixing different ratios of 100% RBC lysate (%L = 100%) and saline. %L = 100% corresponds to ~2.5 μM hemoglobin in the reaction cell. **(B)** Time courses from 0 to 5 s, representing the same records as in **(A)**. **(C)** Dependence of the rate constant of the pH change on the relative amount of RBC lysate. We obtained *k*_ΔpH_-values from non-linear least-squares curve fits of data like those in **(A,B)**. **(D)** Effect of acetazolamide on time courses of pH from 0 to 200 s for %L ranging from 0 to 100%. These experiments are like those in **(A)**, except that we added 10 μM ACZ to solution B (Table 1). **(E)** Time courses from 0 to 5 s, representing the same records as in **(D)**. **(F)** Dependence of *k*_ΔpH_ on %L in the presence of ACZ. Each data point represents mean ± *SD* (error bar not shown if smaller than the symbol). *N* represents the number of independent experiments, each covering six levels of percent RBC lysate (%L: 0–100%).

Second, we extend our work to murine RBC lysates to determine if our CA assay can quantitate hemolysis. The pyranine dye is in the syringe opposite to the one containing RBC lysate (Table 1). Figures 3A,B shows six pH trajectories, similar to those in Figures 2A,B except that here (Figures 3A,B) we replace bCAII with lysate from WT mouse RBCs. A percent lysate (%L) of 100% refers to hemolysate containing 2.5 μM Hb (i.e., Hct ≅0.15%) in the SF reaction cell; a %L of 50% refers to half this concentration of hemolysate (diluted in saline), and so on. In the absence of RBC lysate (i.e., %L = 0% in Figures 3A,B), pH rises from ~7.25 to reach ~7.50 at a low rate that is similar to what we saw above in Figures 2A,B. Here in Figures 3A,B, we see that increasing %L greatly speeds the equilibration of pH. Figure 3C summarizes the best-fit *k*_ΔpH_ data from a total of nine mice. As expected, *k*_ΔpH_ has a linear dependence on %L, and the y-intercept of ~0.0183 s^−1^ is nearly identical to the corresponding value in Figure 2C.

Figure 3D through *F* show that ACZ, at a [ACZ]_RxCell_ of 5 μM, almost completely eliminates the CA activity of the hemolysate.

### CA assay on mixtures of intact and hemolyzed mouse RBCs

**Figure 4:**
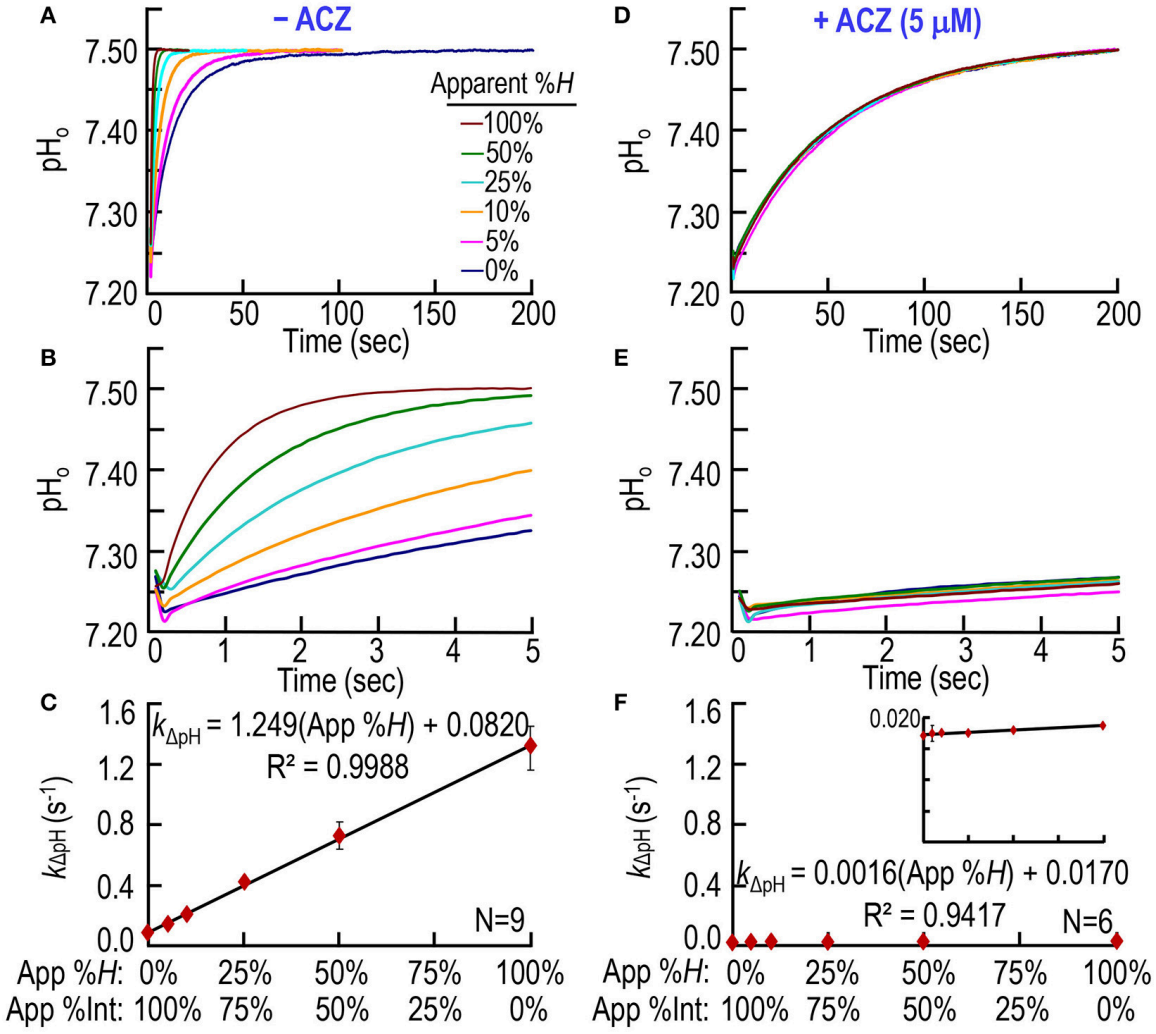
**CA assays on mixtures of intact and hemolyzed RBCs. (A)** Time courses of pH from 0 to 200 s, for degrees of apparent percent hemolysis ranging from 0 to 100%. We added the hemolysate/RBC mixture to solution A (Table 1) and derived pH-values from the fluorescence of pyranine, added to solution B. We created all solutions by mixing different ratios of 100% hemolysate and ostensibly 100% intact RBCs, so that the hemoglobin concentration in the reaction cell was always ~2.5 μM. **(B)** Time courses from 0 to 5 s, representing the same records as in **(A)**. **(C)** Dependence of the rate constant of the pH change on apparent percent hemolysis (App %*H*), which is inversely related to apparent percent intact RBCs (App %Int). We obtained *k*_ΔpH_-values from non-linear least-squares curve fits of data like those in **(A,B)**. **(D)** Effect of acetazolamide on time courses of pH from 0 to 200 s for apparent percent hemolysis ranging from 0 to 100%. These experiments are like those in (A), except that we added 10 μM ACZ to solution B (Table 1). **(E)** Time courses from 0 to 5 s, representing the same records as in **(D)**. **(F)** Dependence of *k*_ΔpH_ on apparent %*H* (inversely related to apparent percent intact RBCs) in the presence of ACZ. Each data point represents mean ± *SD* (error bar not shown if smaller than the symbol). *N* represents the number of independent experiments, each covering six hemolysis/RBC mixtures (App %*H*: 0–100%, App %Int: 100–0%).

In a third set of experiments, we test our CA assay by creating a solution A (Table 1) in which we combine, in different proportions: (a) freshly prepared, ostensibly “intact” RBCs (5 μM Hb, or ~0.3% Hct) with (b) a lysate from an equivalent mass of RBCs (5 μM Hb). Thus, in the SF reaction cell, after mixing with solution B, all solution A combinations generate a Hb concentration ([Hb]_RxCell_)—representing the sum of Hb both inside and outside the RBCs—of 2.5 μM. Thus, this approach simulates ostensible degrees of hemolysis between 0 and 100%, inclusive. Note that the total CA activity in the reaction cell—representing the sum of CAs both inside and outside the RBCs—is also constant across all RBC/lysate mixtures. The pyranine dye is in syringe B, opposite to the one containing the RBC/lysate mixture (Table 1). Figures 4A,B show six pH trajectories obtained on blood from one mouse, and indicate that the greater the ratio of simulated RBC hemolysis the faster the equilibration of extracellular pH (pH_o_).

Note that an ostensibly 0% hemolysis (lowest pH records in Figures 4A,B) produces an equilibration that is considerably faster than the truly uncatalyzed pH trajectories (i.e., the lowest pH records) in Figures 2A,B, 3A,B. Thus, ostensibly 100% intact RBCs must, in fact, be partially hemolyzed in the SF reaction cell. Figure 4C summarizes the best-fit *k*_ΔpH_ data from a total of nine mice—the same nine mice as in Figure 3C. The *k*_ΔpH_ vs. percent hemolysis relationship is linear, as in Figures 2C, 3C. However, in Figure 4C, the y-intercept—the *k*_ΔpH_ of ostensibly 100% intact RBCs from WT mice—is ~0.0820 s^−1^. This value is more than 4-fold higher than the corresponding *k*_ΔpH_ in Figures 2C, 3C, confirming the partial lysis of these cells in the SF reaction cell.

Figures 4D,E show that ACZ, added to the syringe opposite to the one containing the RBC/lysate to achieve a [ACZ]_RxCell_ of 5 μM, almost completely eliminates the CA activity of the mixtures, across the entire range of simulated ostensible hemolysis. Figure 4F shows that, for all mixtures, ACZ reduces *k*_ΔpH_ to nearly the uncatalyzed value. The y-intercept in Figure 4F, which represents the near-fully uncatalyzed state, is only ~21% of the value observed in Figure 4C. Because the pyranine dye and the ACZ first make contact with the RBC/lysate in the SF reaction cell, the dye presumably reports—and the ACZ presumably slows—only the pH equilibration in the extracellular space, and not inside intact RBCs. Thus, the y-intercept in Figure 4C (which represents *k*_ΔpH_ for the uncatalyzed reaction + CA from lysed RBCs) and the y-intercept in Figure 3C (which represents *k*_ΔpH_ for the uncatalyzed reaction only) provide the information needed to compute hemolysis in the SF cell.

### Assessing RBC hemolysis before and during SF experiments

**Figure 5:**
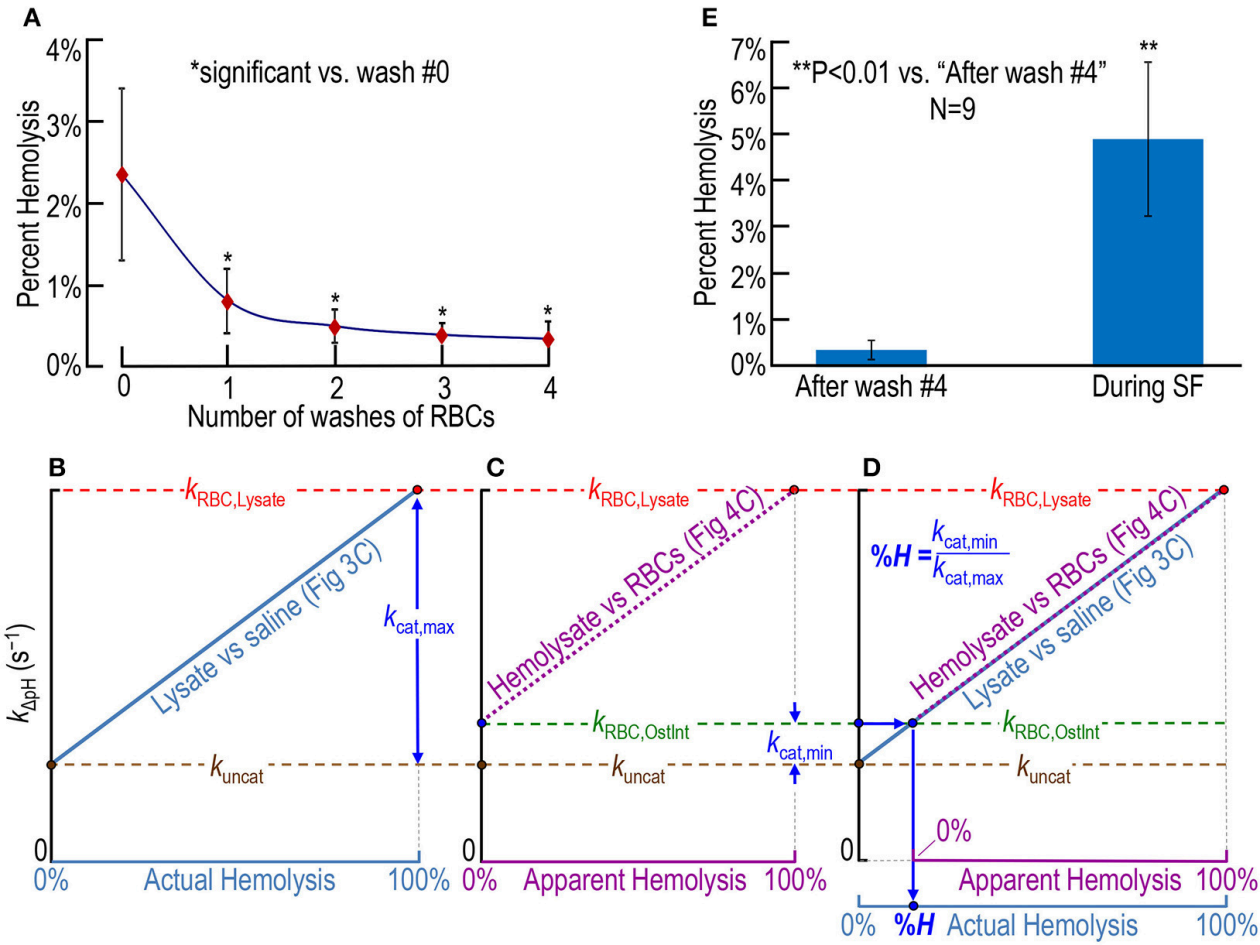
**Assessing RBC hemolysis. (A)** Estimation of the effect of RBC washes (before stopped-flow experiments) on hemolysis, starting with whole blood containing ostensibly 100% intact RBCs. Our approaches for computing percent hemolysis before the first wash or after wash #*i* (*i*: 1–4) are described in Section Materials and Methods under “Blood collection.” **(B–D)** Calculation of actual percent hemolysis of ostensibly 100% intact RBCs during a SF experiment. In **(B)**, we plot the dependence of the rate constant of pH relaxation on actual hemolysis, the latter being represented by increasing mixing ratios of RBC lysate to saline in Figure 3C. Here, *k*_uncat_ represents the uncatalyzed rate constant (i.e., *k*_ΔpH_ without CA), *k*_RBC, Lysate_ represents *k*_ΔpH_ in the presence of 100% lysate [with (hemoglobin) ≅ 2.5 μM], and *k*_cat, max_ represents the portion of *k*_RBC, Lysate_ due to the maximally catalyzed CA reaction. That is, *k*_cat, max_ = *k*_RBC, Lysate_ – *k*_uncat_. In **(C)**, we plot the dependence of *k*_ΔpH_ on apparent hemolysis, the latter being represented increasing mixing ratios of RBC lysate to ostensibly 100% intact RBCs in Figure 4C. Here, *k*_RBC, OstInt_ represents *k*_ΔpH_ in the presence of ostensibly 100% intact RBCs, and *k*_cat, min_ represents the portion of *k*_RBC, OstInt_ due to the minimally catalyzed CA reaction (i.e., that due to the small amount of CA activity released from hemolyzed RBCs in the population of ostensibly 100% intact RBCs). That is, *k*_cat, min_ = *k*_RBC, OstInt_ − *k*_uncat_. In **(D)**, we overlay the plots in **(B,C)**. The percent hemolysis (%*H*) of RBCs during an SF experiment is the quotient *k*_cat, min_/*k*_cat, max_. One may obtain the same answer graphically by following, first, the horizontal arrow from the *y*-axis and, then, the vertical arrow to %*H*. **(E)** Comparison of %*H* of ostensibly 100%-intact RBCs after four washes (i.e., before the SF experiment) and during SF experiments. The value for “after wash #4” in **(E)** is the same as for *i* = 4 in **(A)** (nine mice). The value for “During SF” in **(E)** is the mean for the same nine mice. For each mouse, we obtained plots that were the equivalent of those in **(B)** through **(D)**, and computed a %*H* for that mouse. Each data point represents the mean ± *SD* (lower error bar not shown if it overlaps with *x*-axis tick mark). Differences are evaluated using the two-tailed paired student's *t*-test. In **(A)**, *indicates statistical significance—after applying the Holm-Bonferroni correction—for comparisons to wash #0 of wash #1 (*P* = 0.0013), wash #2 (*P* = 0.00016), wash #3 (*P* = 8.4 × 10^−5^), and wash #4 (*P* = 7.2 × 10^−5^). None of the other comparisons in (**A)** yielded statistical significance. *N* represents the number of mice from which we obtained blood.

In a fourth set of experiments, we collect fresh whole blood from nice mice—the same nice mice as in Figures 3C, 4C—and obtain an initial hemolysis of 2.36 ± 1.04% before any washing (Figure 5A, number of washes = 0). We then wash the RBCs four times in RBC washing solution (Table 1), obtaining the percent hemolysis after each step. After the fourth wash, we arrive at a hemolysis of 0.37% ± 0.21% (which is significantly >0; *P* = 0.00013) before putting the RBCs into the SF device (Figure 5A). Our %*H*-value of 0.37%, which may slightly underestimate the true value as noted in Section Materials and Methods, is similar to the level of 0.5% reported by Itada and Forster in their ^18^O study (Itada and Forster, 1977).

Figures 5B–D show our approach for determining RBC hemolysis in the SF reaction cell. We first determine the relationship between *k*_ΔpH_ and the percent actual hemolysis in an experiment like that in Figure 3C, in which we combine RBC lysate with saline in various proportions. The ascending light blue line in Figure 5B is the idealized result of such an experiment, and leads to two of the three rate constants needed for the calculation of %*H*. The brown circle at 0% actual hemolysis (i.e., 100% pure saline), represents the uncatalyzed rate constant (*k*_uncat_) as the overall reaction ${\text{HCO}}_{3}^{-}$ + H^+^ → CO_2_ + H_2_O approaches equilibrium under the conditions of our experiments. The red circle at 100% actual hemolysis (i.e., 100% RBC lysate), represents the rate constant (*k*_RBC, Lysate_) of the overall dehydration reaction, both uncatalyzed and catalyzed by the all of the CA released from our sample of RBC lysate (i.e., the [CA] corresponding to an [Hb] of ~2.5 μM). The difference between *k*_RBC, Lysate_ (horizontal red dashed line) and *k*_uncat_ (horizontal brown dashed line) represents the rate constant (*k*_cat, max_) of that portion of the overall reaction catalyzed by all the CA present in our sample of RBCs.

The ascending dashed magenta line in Figure 5C is the idealized result of an experiment like that in Figure 4C, in which we combine ostensibly intact RBCs with pure RBC lysate in various proportions. Figure 5C leads to the third rate constant needed for the calculation of percent hemolysis. The green circle at 0% apparent hemolysis (i.e., 100% ostensibly intact RBCs), represents the rate constant (*k*_RBC, OstInt_) of the overall dehydration reaction, both uncatalyzed and catalyzed by the CA released from the small fraction of RBCs that—unbeknownst to us—are hemolyzed at the time the material is inside the SF reaction cell. The difference between *k*_RBC, OstInt_ (horizontal green dashed line) and *k*_uncat_ (horizontal brown dashed line) represents the rate constant (*k*_cat, min_) of the portion of the overall reaction catalyzed by that small amount of CA released from ostensibly intact RBCs.

Finally, in Figure 5D—a superimposition of Figures 5A,B, we see the graphical approach for computing the percent actual hemolysis in the SF reaction cell. Starting from the blue circle on the *y*-axis, we move to the right (blue arrow) until we reach the origin of the magenta dashed line, and then move downward (blue arrow) to read the percent actual hemolysis off the light blue *x*-axis. Mathematically, this value is simply *k*_cat, min_/*k*_cat, max_. Using the approach in Figures 5B–D, we calculate that, even if we start with washed RBCs that are only ~0.37% hemolyzed before they enter the SF device (see above), the total hemolysis is 4.93% ± 1.67% (*SD*) in the SF reaction cell at the end of the experiment (Figure 5E). Thus, the process of performing the SF experiment—loading the RBC mixture into the SF syringes, the flow down the tubing, and the rapid mixing in the SF reaction cell—increases hemolysis.

### Comparing RBC hemolysis using fluorescence vs. absorbance spectroscopy

**Figure 6:**
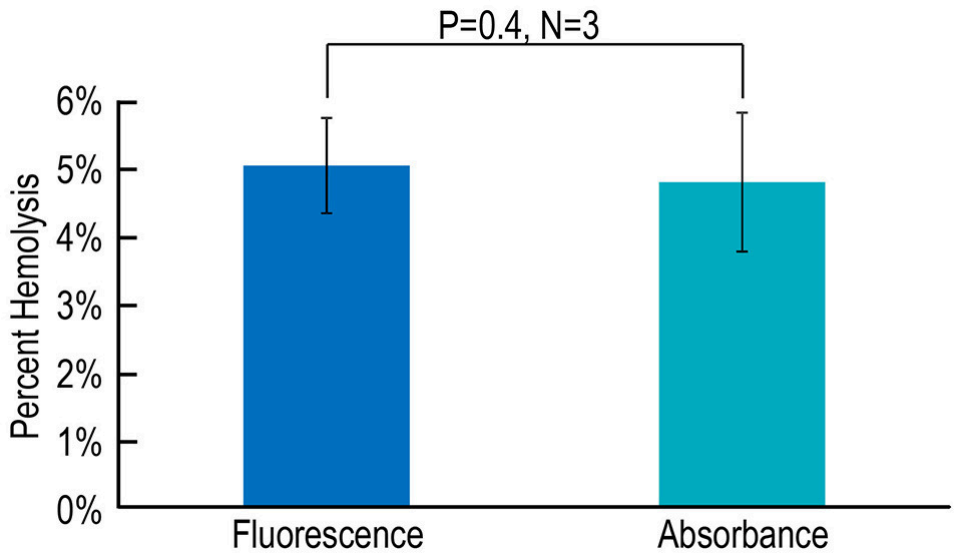
**Assessing hemolysis by fluorescence vs. absorbance spectroscopy**. Each *N* represents blood from one mouse, analyzed in parallel using both a fluorescence-based assay with pyranine and an absorbance-based assay with phenol red. Values are means ± *SD*, evaluated using a two-tailed paired student's *t*-test.

Lastly, we evaluate an alternative approach in which we replace stopped-flow fluorescence spectroscopy (using the dye pyranine) with stopped-flow absorbance spectroscopy (using the dye phenol red). We compare the CA-assay results—fluorescence vs. absorbance—for hemolysis on blood from three mice. As summarized in Figure 6, we find that the estimate of % hemolysis using the fluorescence approach with pyranine is indistinguishable from that that using the absorbance approach with phenol red.

## Discussion

### Comparison of the present CA assay with previous kinetic studies

In the present paper, we describe a simple and rapid assay—based upon the release of CA from RBCs—for determining the degree of hemolysis of RBCs in a SF reaction cell. Moreover, the assay occurs over a time frame and under conditions that are very similar to those of other parallel experiments that generate SF data that are sensitive to percent hemolysis. These other parallel experiments include not only measurements of Hb-O_2_ saturation, but also other assays in which the release of a material (e.g., a pH-sensitive dye) previously inside the cell would confound the measurement. Our search of the literature does not identify previous methods for assessing %*H* in a SF reaction cell. Others have used the time course of light transmittance (a measure of light scattering) to assess relative hemolysis in assays of the osmotic fragility of RBCs (Didelon et al., 2000; Paździor et al., 2003; Górnicki, 2008). In this earlier approach, one can monitor slowly developing (over tens of seconds) changes in transmittance due to progressive hemolysis, but cannot assign a value to hemolysis *per se*.

In the present study, we obtain a single value of hemolysis measured over several tens of seconds, but cannot directly address the time course of hemolysis *per se*. However, because the time courses of pH in Figures 4A,B are exponential beginning no later than ~0.3 s after the stoppage of flow, we can conclude that the hemolysis was virtually complete by this time. In other words, the degree of hemolysis that we measure (right bar Figure 5E) presumably reflects the aggregate hemolysis that occurs to some extent in the preparation of the blood (left bar Figure 5E) but mainly in the syringes, tubing, and the first moments in the reaction cell of the SF device.

Others have previously assessed CA activity using pH-based SF approaches (Gibbons and Edsall, 1963, 1964; Ho and Sturtevant, 1963; Kernohan, 1964, 1965; Khalifah, 1971; Wistrand et al., 1975; Pocker and Bjorkquist, 1977; Crandall and O'Brasky, 1978; DeGrado et al., 1982; Sanyal et al., 1982; Baird et al., 1997; Shingles and Moroney, 1997; Wang et al., 2010) with tissue homogenates or cell-free enzyme preparations. The ionic conditions in these earlier studies would have been inappropriate for living cells. By definition, these earlier studies involved monitoring pH as the solution in the SF reaction cell transitioned from an OOE state toward an equilibrium state. However, fundamental differences between our approach and those of others are that the present assay provides:

1. Solutions A and B of moderate and defined pH-values. Regarding the previous SF studies of CA activity, only one (Shingles and Moroney, 1997) reports SF time courses of pH *per se*, and only one (Shingles and Moroney, 1997) provides sufficient detail (including pH-values of both solution A and solution B) to permit replication of the work. In our assay, both solution A (pH 7.03) and solution B (pH 8.41) have relatively moderate pH-values, thereby ensuring that the RBCs are under near-physiological electrolyte conditions throughout the assay. Even though the CA enzyme or RBCs are in the near-neutral solution A in our assay, it is important that solution B not have an extreme *p*-value. Earlier OOE work (Zhao et al., 1995) provided evidence of transient microdomains upon mixing of solutions A and B (i.e., the enzyme or cells in solution A could come into direct contact with relatively undiluted solution B). In principle, we could have designed a solution B with a lower pH. A consequence (all else being equal) would have been a smaller ΔpH during the relaxation phase of the experiment. However, the signal-to-noise resolution of our assays was sufficiently high that we could have still obtained reliable *k*_ΔpH_ data, even with a smaller ΔpH.
2. Predictable pH-values in the SF reaction cell. Our OOE approach generates, upon mixing of solutions A and B, predictable pH-values at time zero, and predictable pH time courses (simulations not shown).

Although the assay in the present study is based on the reaction $\text{HC}{\text{O}}_{3}^{-}+{\text{H}}^{+}\overset{\text{CA}}{\to }\text{C}{\text{O}}_{2}+{\text{H}}_{2}\text{O}$ (equivalent to the dehydration of H_2_CO_3_) and thus produces a pH increase, we could have constructed solutions A and B so that, upon solution mixing in the reaction cell, the OOE state would have led to the opposite reactions (equivalent to the hydration of CO_2_) and thus a pH decrease.

### Experiments at 10°C

Transport events across the RBC membrane can be extremely fast. In the present study, we chose 10°C to match the temperature in parallel work on O_2_ fluxes. This temperature, although considerably less than typical physiological temperatures of mammals, is nevertheless higher than those used by other investigators in RBC-transport studies, where it is customary to lower RBC temperatures to 5°C or even 0°C (Kimzey and Willis, 1971; Lowe and Walmsley, 1986; Jensen et al., 2001; Jennings, 2005).

### CAs in RBCs

The 14 mammalian α CAs (Sly and Hu, 1995; Purkerson and Schwartz, 2007; Supuran, 2016) are zinc-containing metalloenzymes that are expressed in virtually every cell of the body. They are broadly divided into four subgroups: the cytosolic [a] CA I, II, III, VII, and VIII; [b] the mitochondrial CA V; [c] the secreted CA VI; and [d] the membrane-bound CAs IV, IX, XII, and XIV (Sly and Hu, 1995; Parkkila et al., 1996; Karhumaa et al., 2000; Kummola et al., 2005; Scheibe et al., 2006). A proteomics study identified CA I, II, and III in RBC cytoplasm (Kakhniashvili et al., 2004). In human erythrocytes, CA II is a high-activity isozyme; CA I contributes only half of the total CA activity although it is almost six times as abundant as CA II (Khalifah, 1971; Dodgson et al., 1988; Sly and Hu, 1995). CA III—the activity of which is reported to be only 0.03–1% that of CA II (Sly and Hu, 1995; Purkerson and Schwartz, 2007)—is expressed mainly in skeletal muscle and adipose tissue, but at lower levels in other tissues, including RBCs (Sly and Hu, 1995; Kakhniashvili et al., 2004; Pasini et al., 2006; Goodman et al., 2007). Within the RBC, these CA enzymes are critically important for converting metabolically produced $\text{C}{\text{O}}_{2}\text{ to HC}{\text{O}}_{3}^{-}(\text{C}{\text{O}}_{2}+{\text{H}}_{2}\text{O}\overset{\text{CA}}{\to }\text{HC}{\text{O}}_{3}^{-}+{\text{H}}^{+})$ while the RBC is in systemic capillaries, and then—after the RBC has transited to the lung—for reconverting the $\text{HC}{\text{O}}_{3}^{-}\text{ to C}{\text{O}}_{2}(\text{HC}{\text{O}}_{3}^{-}+{\text{H}}^{+}\overset{\text{CA}}{\to }\text{C}{\text{O}}_{2}+{\text{H}}_{2}\text{O})$ in the pulmonary capillaries for elimination in the exhaled air.

Although a mass-spectrometry analysis study reveals no evidence of membrane-associated CAs in RBCs (Low et al., 2002), immunological and kinetic evidence points to a tiny amount of CA IV, presumably on the outer surface, that could contribute 0.2% of total CA activity (Wistrand et al., 1999). If this CA IV estimate for RBCs is correct, then 0.2/4.93% ≅ 4% of the estimated %*H* of 4.93% (Figure 5E) in the SF reaction cell could reflect the presence of extracellular-facing CA IV rather than hemolysis *per se* (i.e., we may slightly overestimate the degree of hemolysis).

### Evidence for the validity of the present CA assay

The following analyses of our data provide evidence that our CA assay behaves in a stable and precise way:

1. *Linear relationship of k*_Δ*pH*_ *vs. [bCAII]*. For the experiments in Figure 2, we chose a commercial preparation of bovine CA II, purified from RBCs, because bCAII is the most readily obtainable and reliable CA preparation. As expected, *k*_ΔpH_—which reflects the equilibration of all reactions as pH rises from ~7.25 to 7.50—rises linearly with increasing concentrations of CA II (Figure 2C). Thus, we hypothesized that we could exploit the release of CAs from RBCs to quantitate the degree of hemolysis.
2. *Linear relationship of k*_Δ*pH*_ *vs. percent lysate*. For the experiments in Figure 3, we chose to work with the lysate of a preparation of mouse RBCs having a [Hb] of ~2.5 μM in the reaction cell—the starting material for the ostensibly intact RBCs in Figure 4. As expected, *k*_ΔpH_ in Figure 3C rises linearly with increasing %L (Figure 3C), reflecting the increasing concentration of CAs.
3. *Linear relationship of k*_Δ*pH*_ *vs. percent hemolysis*. For the experiments in Figure 4, we dilute ostensibly intact RBCs with pure RBC lysate to maintain a [Hb] of ~ 2.5 μM in the reaction cell. Note that pyranine [a] permeates cell membranes poorly (Shingles and Moroney, 1997; Avnir and Barenholz, 2005; Han and Burgess, 2010) and [b] is in contact with the RBCs for only a few seconds. We find that pH_o_ rises with a near-exponential time course in Figures 4A,B, and that the resulting *k*_ΔpH_-values rise linearly with increasing %*H* (Figure 4C). If a substantial fraction of total pyranine were slowly entering RBCs (which undergo a pH decrease as CO_2_ enters and ${\text{HCO}}_{3}^{-}$ exits via AE1), the pH_o_ trajectory would have had a second phase that reflected the evolving contribution of the pH inside the RBCs. Thus, we can conclude that the CA inside the RBC is not appreciably accessible to the dye under the conditions of our experiments.
4. *Consistency of 100% lysate/hemolysis points in* Figures 3C, 4C. In Figure 3C, the best-fit value for *k*_ΔpH_ when %L = 100% is 1.304 × 100% + 0.0183 = 1.322 s^−1^. In Figure 4C, the comparable value for *k*_ΔpH_ when %*H* = 100% is 1.249 × 100% + 0.0820 = 1.331 s^−1^. These two values differ by < 0.7%, indicating the internal consistency of the two data sets.
5. *CA activity of mouse RBCs*. From the previous paragraph, we see that the *k*_ΔpH_ in the 100% mouse RBC lysate (Figure 3C) is 1.322 s^−1^. From the line of best fit in Figure 2C, we see that a mouse RBC lysate value of 1.322 s^−1^ corresponds to a [bCAII]_RxCell_ of 1.6 μg/ml. Given a MW of 29,000 Da for bCAII, we calculate that the total CA activity (from mouse RBCs) in the reaction cell (representing 100% lysate) is equivalent to that of ~0.055 μM bCAII. Although we set mouse [Hb]_RxCell_ to 2.5 μM in Figure 3C, the mean corpuscular hemoglobin concentration (MCHC) for wild-type mouse RBCs that we obtained in a parallel study is 31.7 g/dL, which corresponds to a [Hb]_i_ of ~4.9 mM. Thus, the [Hb] in the reaction cell of the present study represents a ~1967-fold dilution of cytosolic Hb. We calculate that the total CA contributed by the mouse RBCs behaves as if it were ~109 μM bCAII. We are not aware of data on the CA content of mouse RBCs. However, the mean of three values for the CAII/Hb ratio of human RBCs (Tashian and Carter, 1976; Ali Akbar and Brown, 1996), together with an assumed [Hb]_i_ of 5 mM, leads to [CAII]_i_ of ~20 μM for human RBCs. Thus, our computed CA activity of mouse RBCs (equivalent to ~109 μM bCAII) is ~5-fold higher than the [CAII]_i_ of human RBCs. Considering that CAII contributes only about half of the total CA activity of human RBCs, and that the purchased bCAII may have a lower specific activity than the total CA released from freshly lysed mouse RBCs, we conclude that the equivalent concentration of ~109 μM bCAII is reasonable.
6. *Consistency of slopes in* Figures 3C, 4C. As illustrated by the analysis in Figures 5B–E, the difference between the slopes of the best-fit lines in Figure 3C (i.e., 1.304 s^−1^/%L) and Figure 4C (i.e., 1.249 s^−1^/%*H*) is almost completely accounted for by the actual %Hemolysis of ostensibly intact RBCs (i.e., 4.93%). That is, 1.304 × 95.07% = 1.240, which is only ~0.7% lower than 1.249.
7. *Comparison of y-intercepts in* Figures 3C, 4C. In Figure 3C, the best-fit value for the y-intercept (i.e., %L = 0%) is 0.0183 s^−1^, which is the rate constant for the uncatalyzed reactions. In Figure 4C, the comparable best-fit y-intercept is 0.0820 s^−1^, which is the aggregate *k*_ΔpH_ for the uncatalyzed and catalyzed reactions. The difference 0.0820 s^−1^ − 0.0183 s^−1^ = 0.0637 s^−1^, which represents the mean portion of *k*_ΔpH_ attributable to the extracellular CA activity of ostensibly intact RBCs. Dividing this residual y-intercept by the best-fit slope in Figure 3C yields the mean %*H* of ostensibly intact RBCs: (0.0637 s^−1^)/(1.304 s^−1^/%L) = 4.9%. Performing the computation mouse by mouse, we obtain 4.93 ± 1.67% (*N* = 9). The value, compared to the pre-SF value of 0.37 ± 0.21% indicates that a small amount of ostensibly intact RBCs are in fact hemolyzed in the SF reaction cell.
8. *Effect of ACZ in* Figures 2D–F, 3D–F, 4D–F. In all of our studies, ACZ collapsed all pH trajectories to virtually the same time course—and the same pH range—as the uncatalyzed (i.e., slowest) reactions in Figures 2A–C, 3A–C. However, we notice that the best-fit y-intercepts in Figures 2F, 3F, 4F are all very slightly lower than those in Figures 2C, 3C. An analysis of the [bCAII]_RxCell_ = 0 points in Figure 2C (*k*_ΔpH_ = 0.0185 ± 0.0014 s^−1^) and Figure 2F (*k*_ΔpH_ = 0.0170 ± 0.0005 [*SD*] s^−1^) is a statistically significant difference (*P* = 0.028, two-tailed, unpaired *t*-test). For the %L = 0 points in Figure 3C (*k*_ΔpH_ = 0.0183 ± 0.0019 s^−1^) and Figure 3F (*k*_ΔpH_ = 0.0176 ± 0.0007 s^−1^), the difference is not statistically significant (*P* = 0.37, two-tailed, unpaired *t*-test). For the %*H* = 0 point in Figure 4F, *k*_ΔpH_ = 0.0170 ± 0.0006. Although it is possible that ACZ affects the pyranine dye, we suggest that it is more likely that, despite extensive washing between samples, a small amount of CA remains adsorbed to the surface of the reaction cell. Such a small amount of adsorbed CA would not affect our calculation of %*H* of ostensibly intact RBCs from a mouse because the uncatalyzed *k*_ΔpH_-value that we subtract is the value obtained in %L = 0 from an experiment like that in Figure 3B (not the one with ACZ in Figures 3E or 4E).
9. *Phenol red vs. pyranine in* Figure 6. We observed that our novel CA/hemolysis assay—applied to the same murine RBC samples—yields virtually identical results with two different dyes, one studied by stopped-flow fluorescence and the other by stopped-flow absorbance spectroscopy. The absorbance-spectroscopy method with a non-fluorescent dye would be particularly useful when the system contains materials with significant fluorescence.

## Conclusion

Our novel CA assay with OOE solutions makes it possible to assess hemolysis under approximately physiological conditions, and to do so over a predictable pH range. The increase in percent hemolysis between the time of loading samples into the SF machine (< 0.4%) and during our SF assay (~4.9%) presumably due to mechanical disruption of a subpopulation of susceptible RBCs during rapid mixing. This assay is easy to perform, highly sensitive and precise, and in principle could be implemented in either the direction $\text{HC}{\text{O}}_{3}^{-}+{\text{H}}^{+}\overset{\text{CA}}{\to }\text{C}{\text{O}}_{2}+{\text{H}}_{2}\text{O}$ (equivalent to H_2_CO_3_ dehydration, as in the present study) or the direction $\text{C}{\text{O}}_{2}+{\text{H}}_{2}\text{O}\overset{\text{CA}}{\to }\text{HC}{\text{O}}_{3}^{-}+{\text{H}}^{+}$ (equivalent to CO_2_ hydration). Moreover, it is readily amenable to miniaturization and automation using microfluidics. Applied to ostensibly intact RBCs, the assay could report RBC fragility and thus provide diagnostic insight in fresh blood from patients. Besides RBCs, the lysis assay could detect disruption within a SF device of many other cell types or membrane vesicles with entrapped CA. The underlying CA assay—applied to fluids such as blood plasma, tissue-culture media, and organ perfusates—could assess cell lysis in a wide range of cells or tissues previously in contact with these fluids. Applications could include quantitating RBC storage lesions before performing blood transfusions, evaluating the health of cells in culture or during flow cytometry, and assessing the health of organs for transplantation. Together with a panel of specific CA inhibitors, our assay also could quantitate multiple different CA subtypes within a sample, and identify the lysed cells from which they came.

## Author contributions

PZ and RG have contributed equally to this work. PZ, RG, and WB contributed to experimental design. PZ and RG performed experiments and analyzed data. PZ, RG, and WB wrote the manuscript. All authors approved the final version to be published.

## Funding

This work was supported by grants from the Office of Naval Research (N00014-11-1-0889, N00014-14-1-0716, and N00014-15-1-2060 to WB). RG was supported by a fellowship grant from the Office of Naval Research (N00014-12-1-0326).

### Conflict of interest statement

The authors declare that the research was conducted in the absence of any commercial or financial relationships that could be construed as a potential conflict of interest.

## Footnotes

^1^We assumed that the system is closed for CO_2_/${\text{HCO}}_{3}^{-}$, with initial [CO_2_] = 0.23 mM, initial [${\text{HCO}}_{3}^{-}$] = 22 mM, pK = 6.2, and final pH = 7.50. The difference between the decrement in [${\text{HCO}}_{3}^{-}$] and the increment in [CO_2_] is due to rounding errors.

## Abbreviations

App %*H*: apparent percent hemolysis
App %Int: apparent percent intact RBCs
%*H*: percent hemolysis
%L: percent lysate
*k*_ΔpH_: the rate constant of pH relaxation
*k*_RBC, OstInt_: *k*_ΔpH_ in the presence of ostensibly 100% intact RBCs
*k*_RBC, Lysate_: *k*_ΔpH_ in the presence of 100% lysate
*k*_cat, max_: portion of *k*_RBC,Lysate_ due to the maximally catalyzed CA reaction
*k*_cat, min_: portion of *k*_RBC,OstInt_ due to the minimally catalyzed CA reaction
*k*_uncat_: uncatalyzed rate constant
RxCell: stopped-flow reaction cell.
